# Using systems thinking to increase understanding of the innovation system of healthcare organisations

**DOI:** 10.1108/JHOM-01-2022-0004

**Published:** 2022-06-20

**Authors:** Gary Linnéusson, Thomas Andersson, Anna Kjellsdotter, Maria Holmén

**Affiliations:** School of Engineering , Jönköping University , Jönköping, Sweden; School of Business , University of Skövde , Skövde, Sweden; Research and Development Centre , Skaraborg Hospital Skövde , Skövde, Sweden; Innovation Platform , Region Västra Götaland , Gothenburg, Sweden

**Keywords:** Innovation system, Healthcare organisation, Organisational culture, Systems thinking

## Abstract

**Purpose:**

This paper applies systems thinking modelling to enhance the dynamic understanding of how to nurture an innovative culture in healthcare organisations to develop the innovation system in practice and speed up the innovative work. The model aims to provide a holistic view of a studied healthcare organisation's innovation processes, ranging from managerial values to its manifestation in improved results.

**Design/methodology/approach:**

The study is based on empirical material from a healthcare unit that, within a few years, changed from having no innovations to repeatedly generating innovations. The study uses the modelling language of causal loop diagrams (CLDs) in the system dynamics methodology to identify the key important aspects found in the empirical material.

**Findings:**

The proposed model, based on the stories of the interviewees, explores the dynamics of inertia when nurturing an innovative culture, identifying delays attributed to the internal change processes and system relationships. These findings underscored the need for perseverance when developing an innovative culture in the entrepreneurial phases.

**Practical implications:**

The approach of using systems thinking to make empirical healthcare research results more tangible through the visual notations of CLDs and mental simulations is believed to support exploring complex phenomena to induce and nurture both individual and organisational learning.

**Originality/value:**

The results from this approach provide deepened analysis and provoke the systems view to explain how the nurturing of the culture can accelerate the innovation processes, which helps practitioners and researchers to further expand their understanding of their healthcare contexts.

## Introduction

Progressively understanding the pre-conditions for innovation in healthcare has been acknowledged to be of great importance ( Atun, 2012 ; Savory and Fortune, 2015 ; Thune and Mina, 2016 ) in terms of achieving efficient, safe, high-quality care and, at the same time, meeting the needs of various stakeholders ( Larisch *et al.* , 2016 ), as well as considering global health challenges ( Elias, 2021 ; Haynes *et al.* , 2020 ). Although innovation is considered a critical capability of healthcare organisations ( Savory and Fortune, 2015 ), knowledge about innovation in healthcare is surprisingly scarce ( Øvretveit *et al.* , 2012 ). According to Acar and Acar (2012) and Day-Duro *et al.* (2020) , organisational culture has been identified as one of the most important antecedents of healthcare organisations' innovativeness. Organisational culture has not only been identified as an important condition for healthcare innovations, but also as one of the main blocking mechanisms for innovations ( Keller *et al.* , 2013 ; Mannion and Davies, 2018 ). However, research tends to state that organisational culture *is* important for healthcare innovation rather than *how* it is important. The tendency is to treat culture as current state (see, e.g. Acar and Acar, 2012 ; Carmo Caccia-Bava *et al.* , 2006 ), but treating it as an evolving and processual change, as presented by Day-Duro *et al.* (2020) , would help explain how it can be nurtured and grow over time. Additionally, the active role of healthcare organisations in generating innovations is often undervalued in current research ( Thune and Mina, 2016 ). Consequently, a greater understanding of healthcare organisations' active role in the innovation processes is needed in order to describe how culture as a process can support innovations in healthcare and strengthen the innovation system. Furthermore, innovativeness may differ significantly in the same healthcare organisation ( Exton, 2010 ), a leadership supporting employees by nurturing values is needed ( Day-Duro *et al.* , 2020 ), and adequate resources are a prerequisite to enable working with innovations ( Exton, 2010 ; Larisch *et al.* , 2016 ) as well as, the fundamental aspect of facilitating employees to think in new ways about their work, imperative in systems thinking ( Stalter and Mota, 2018 ).

In line with previous healthcare researchers, the present paper promotes the use of systems thinking, and specifically uses causal loop diagrams (CLDs) (as in, e.g. Best *et al.* (2016) and Elias (2021) ), to address the inherent dynamic complexity of healthcare systems to achieve innovative change towards more efficient, safe and high-quality care. Innovative transformational change requires new ways of thinking, and according to Swanson *et al.* (2012) , applying systems thinking creates preconditions for local health practitioners to innovate in their local hospital unit, having the wider effect of improving community health and thus the overall health system. Moreover, the aim of systems thinking modelling is to impact how we think of our systems offering the larger potential for policy impact ( Haynes *et al.* , 2020 ), inducing learning ( Waldman, 2007 ), and questioning one's mental models ( Senge and Sterman, 1992 ). Such modelling can provide opportunities to promote understanding by allowing explicit learning models for testing and revising our understanding of the nature of things ( Peters, 2014 ).

Consequently, this study has investigated a smaller healthcare unit which successfully had undergone an innovative transformational change in recent years. The stories of the interviewees began to emerge into a systemic structural pattern of *how* nurturing an innovative culture in one end, led to more and more frequent innovation projects in the other, much owing to the unit leader's commitment and strong internal vision. However, to enable reasoning about the driving and hindering forces of the complex dynamics identified from the empirical findings, further mapping using the modelling language of a CLD was applied. Moreover, studying the entirety of the CLD and reasoning on its dynamics can support highlighting the complexity of the identified blocking mechanisms of organisational culture and *how* important nurturing desired *values* is to maintain innovative momentum. Since culture is hard to describe, often partly un-reflected and taken for granted ( Schein, 2017 ), the proposed model may serve as an inspirational tool in other healthcare contexts to expose current unambiguous cultures, or at least to support the process of such doing. The use of a CLD for storytelling to analyse successful innovation systems ( Elias, 2021 ) is believed to allow open and coherent scrutiny of the studied case, despite its limitations, through its explicit presentation of the studied phenomena. This provides value for both the field of healthcare and innovation research, and further usage of the model may also support actors in healthcare organisations to develop new shared understanding of their culture or collective identity ( Bååthe and Norbäck, 2013 ). It is believed that the use of systems thinking to visualise the empirical findings, and analysing the proposed model's possible dynamics, can contribute to the growing body of research on innovative culture in healthcare organisations (see, e.g. Andersson *et al.* , 2019 ; Day-Duro *et al.* , 2020 ; Dopson *et al.* , 2008 ; Exton, 2010 ; Larisch *et al.* , 2016 ; Øvretveit *et al.* , 2012 ).

## Systems thinking to support healthcare innovations

Healthcare systems are characterised by being immensely complex, emerging from the many interconnected and interdependent elements and their feedback structures, including multiple independent agents connected with the surrounding environment. As these systems evolve and self-adapt, they may become even more complex, qualifying as complex adaptive systems ( Peters, 2014 ; Waldman, 2007 ). The interconnected nature of systems, with feedback and delays, gives rise to dynamic complexity and even the seemingly simple dynamic problem has proven difficult for the human mind to comprehend and make decisions on ( Sterman, 2000 ). It requires systems thinking as an appropriate tool to think about complexity in health ( Rusoja *et al.* , 2018 ). Further, systems thinking has been identified as supporting implementing change in health service delivery in implementation science ( Sarkies *et al.* , 2021 ); complementing and enriching the predominant reductionist approaches to health improvement ( Swanson *et al.* , 2012 ); dissolving the dysfunction of repeated unintended consequences from policies in healthcare ( Waldman, 2007 ); supporting the conceptualisation of health problems and health contexts to work effectively with stakeholders ( Haynes *et al.* , 2020 ); and, by studying system functioning as a whole, providing insights to improve healthcare quality ( Leslie *et al.* , 2018 ). It is also considered a precondition in health system resilience research ( Saulnier *et al.* , 2021 ).

Hence, many works propose systems thinking to support innovative thinking of healthcare systems. However, large amounts of literature are often considered to be theoretical ( Rusoja *et al.* , 2018 ), and the number of empirical research and concrete applications is far smaller than the number of calls to apply systems thinking interventions in health systems ( Kwamie *et al.* , 2021 ). Yet, in their practically oriented paper, Stalter and Mota (2018) suggest examples of how nurses can apply systems thinking by elaborating on developing the generic traits of systems thinkers. Some of these traits are, developing an awareness of the studied system, apprehending patterns and structures governing system behaviour and enhancing understanding of how system relationships can be linked to system improvements, which together build confidence and willingness to challenge current systems and problem boundaries despite existing hierarchies. All contribution to nurses under such training will be better equipped to nurture development in organisations to envision quality and safety in healthcare. Consequently, the traits of a systems thinker are directly transferable and worth increasing to understand any complex system, such as the innovation system of healthcare organisations.

The system dynamics methodology provides a systems language to study and identify the interconnections between parts in relevant system boundaries to gain a better understanding of reality ( Forrester, 1961 ; Sterman, 2000 ). To study dynamically complex problems, one can use qualitative modelling (CLDs) to represent the feedback structures, and use quantitative modelling, and adding stocks and flows, which besides feedback structures represent the fundamental building blocks of dynamic systems ( Forrester, 1961 ). A CLD uses mental simulation, while a quantitative stock-and-flow diagram uses computer simulation. There are considerable applications of these systems thinking tools in healthcare; for examples, see recent reviews by Chang *et al.* (2017) , Chughtai and Blanchet (2017) , Rusoja *et al.* (2018) and Wilkinson *et al.* (2018) . Furthermore, simulation can support studying the trade-offs between short- and long-term dynamics ( Linnéusson *et al.* , 2018 ), calculate cost consequences from healthcare policies ( Linnéusson and Goienetxea Uriarte, 2021 ) or enable multi-objective optimisation to extract higher order learning from the behavioural landscape of a model ( Linnéusson *et al.* , 2020 ). Nevertheless, the goal of using these tools, qualitative as quantitative, is to provoke novel ideas and potentially stimulate change in system actor's mental models ( Senge and Sterman, 1992 ), facilitate learning and increase levels of shared mental models (SMMs) to integrate across inter-organisational and inter-professional boundaries ( Evans and Baker, 2012 ), and helping to create a process to uncover unaware aspects of the studied problem as well as unknown unaware aspects resulting in a deeper individual understanding of the studied phenomena ( Holmström *et al.* , 2021 ).

## Methods

The case study was conducted at a speech therapy unit at a medium sized hospital in Sweden, as part of a larger multi-case research programme with the purpose of mapping and analysing the innovation system of a hospital. The studied unit was selected due to its recent and successful increase in its innovative capabilities, supported by the evolvement of the organisational culture, making the culture change more prominent for both its members and the observing researchers. The study design and sample consisted of a qualitative case study approach using semi-structured interviews with 13 women and one man from various professions, such as speech therapists, nurses, managers and physicians. The data collection followed using an interview guide with few open-ended questions where the informants were encouraged to narrate about their experiences of events behind successful innovations and what the preconditions could have been, asking questions such as the following: “Can you tell us about yourself?”, “Can you tell us about the experiences concerning your innovative working?”, “What role have you played in the project and what is your role today?” and “Can you describe the process of the project from the beginning to where it is today”. Thus, the different interviews provided different data of the innovation system to help understand the more complete overall phenomena and procedural changes ( Day-Duro *et al.* , 2020 ), rather than on understanding specific innovations. Similar to Day-Duro *et al.* (2020) , we define innovation in healthcare in its broadest sense, encompassing the development or refinement of any new process, product or delivery method. Innovations that had taken place at the studied unit considered, e.g. a new planning system to minimise late and costive cancellations by patients, introducing digital-based care programmes to better succeed with required exercises between patient interventions to increase quality. The interviews were recorded; four were conducted at the hospital, while the rest were conducted digitally face-to-face, due to the COVID-19 pandemic. All of the interviews were conducted between March and December of 2020 and lasted around 60 min each. Regarding the ethical considerations, principles in the Helsinki Declaration ( WMA, 2008 ) were followed, where participants orally obtained information on the research aim, that it was voluntary to participate, and that they could withdraw from the study at any time, and all interviewees were guaranteed that the data would be treated confidentially.

The data analysis of the interview texts used thematic analysis, following Braun and Clarke (2006) , which implied the six phases: familiarise, generate initial codes, develop themes, review potential themes, define and name themes, and produce the report. All phases were developed through continuous discussions amongst the researchers to strengthen the consistency of the findings. The thematic analysis was also supported by previous research on innovative culture in healthcare organisations, together with a theoretical framework for analysing culture. Analysing the empirical findings was characterised by an iterative process between the entire data set, the coded meaningful pieces of texts and ongoing data analysis. In the end phase of the thematic analysis, as it was noticed many of the pieces of findings were interconnected in networks hard to overview; the researchers began mapping the findings using CLDs. A first prototype CLD was developed to create a comprehensible map over important variables and how these could interrelate. An exploration of how the hypotheses of how to represent the empirical findings could be, together with reasoning about the model's explanatory power to visualise the nurturing of an innovative culture. This iterative verification process aimed to assure that the attributes of the initial codes and potential themes were represented and that the assumptions of the CLD to a satisfying degree explained our perceptions of the empirical findings on how variables could be connected and hold true to this aim. Hence, the model developed in a set of cycles with the research team, where the different views of the pieces of the empirical findings could be integrated in a meaningful whole in the CLD. Each cycle led to, not only completing the CLD, but also to finalising the codes and themes. Having composed and verified the CLD within the research team the next step was to compile a deeper explanation of it, as presented herein, to detail its construct and subsequently analyse the emerged model composition even further to explore the entirety of having composed the CLD.

Applying a CLD activates mental simulation to enable testing the resulting dynamic hypothesis of an articulated problem, using qualitative reasoning ( Sterman, 2000 ). Hence, both constructing and applying CLD as a method is associated with uncertainty regarding the resulting analysis. Hence, using the systems thinking approach serves mainly the purpose of exploring current perceptions of how dynamically complex phenomena can arise, as well as potentially exploring consequences thereof. It allows for qualitative reasoning on how behaviour is generated or degenerated and potentially evolves on both the short- and long-run, considering the context of the studied problem based on the identified relevant system boundary where a system boundary oftentimes can bridge the common limit of silo thinking by acknowledging aspects across organisations that have significant contributions to problem phenomena ( Wolstenholme, 2004 ), thus supporting decision making on a higher system level, then, otherwise. Moreover, composing a CLD based on the puzzle of interview stories as presented herein naturally contains limitations, as well as the subsequent analysis and conclusions thereof, and need further processing together with stakeholders and problem owners for any future implementation. Additionally, the delimitation of the model boundary here is the scope of the empirical findings which had the purpose to understand the innovation system of healthcare organisations. The above described research approach led to highlighting nurturing an innovative culture as one key finding and to composing a CLD to visualise the interconnectedness of the identified pieces of the interview stories. Subsequently, according to Best *et al.* (2016) , applying system thinking tools such as CLDs can easily be explained and understood if presented stepwise, despite their bewildering appearance as a complete schematic diagram. However, the potentially rewarding educational effect from so doing is not explored within this study. An additional delimitation is the evaluation of utilising the model in workshops and how it may affect the patterns of conversation between those confronted by it, which may induce reflections on their own culture and create a larger awareness of it, to enable deliberate shaping and shared understanding towards the new desired culture ( Bååthe and Norbäck, 2013 ).

## Summary of the empirical basis for the study

The results are presented based on the premise that organisational culture governs people's actions based on shared assumptions and values amongst its members ( Schein, 2017 ). Hence, innovation culture is considered to be inter-tangled with the organisational culture rather than as something separate. The case study is presented using seven themes, which are summarised as follows:

*Values* : The former manager of the unit was said to have been the bearer of the values of growing an innovative culture now manifested, by supporting the current innovation climate and continuous reification of shared values. The manager was also considered to have influenced the dominant ideas and ways of thinking, and being accessible and a good listener.

*Expand the myopic view* : The former manager also expanded the employees' appreciation for competencies beyond their own health profession, such as digitalisation and design thinking, via external networks and external researchers and internal hospital healthcare improvement workers. These aspects allowed for new thinking on old problems, and increased organisational openness and the ability to see the larger picture. This led to seeing both the patients' individual and collective needs, which meant that improvements for groups of patients became a course to improve for the individual patient too.

*Advocating for bottom-up initiatives* : Employees were actively encouraged to act as sensors to identify and think innovation possibilities by the manager, leading to the creation of a decentralised improvements culture. It resulted in a high level of shared values over time and trust manifested in a more open innovation climate, with high legitimacy to think aloud and try new procedures.

*External affirmation of innovative acting* : Employees were encouraged to present their innovations on several occasions and at multiple arenas, both externally and at the hospital. Together with the above processes, it created pride for their work and led to further nurturing their attitudes to innovative work and self-images into being active and taking initiatives.

*Resources to enable reflection* : Giving employees time to reflect on daily work and development projects was seen as a necessity and a hotbed for innovations. The journey had taught the new manager that innovation requires resources to allow for reflection; it does not just happen by itself and must be an integrated task, not something in addition to ordinary work. Success in external funding for healthcare innovations provided additional resources to enable time for conducting innovation projects, which had sped up the process and would have resulted in a much slower process otherwise.

*Managing within short-termism to support values to innovative actions* : Higher-level management focussed on the short-term cost, which resulted in the former manager needing to find spaces within the ordinary budget for innovative work and nurturing the innovative culture undercover. The innovations were applauded, but regarding budget, no interest existed to pay for them. Despite the less developed innovative culture at the hospital level, the unit manager could manifest her values and actions, requiring both courage and experience, and enable the above processes to be acted out at the hospital unit.

*Nurturing leading to sustainable structures* : The case illustrated how nurturing an innovative culture utilised the organisational structures, for instance, in weekly meetings having innovations on the agenda, and how little the structure itself was the enabler. Instead, the enabler was the level of open innovation climate, where employees see themselves as active and initiative taking and feel the trust from their manager. This indicated the intertwined nature of organisational structure and culture. Also, the journey manifested in new structures where one new base criterion for future employees was that they should have an innovative mindset and interest in improvement work.

## Model description

Attributes and stories from the interviews have been transformed into variables and defining how these are interconnected. Most variables are dependent on other variables, and nearly all are connected in multiple coupled relations. The process of creating a CLD involves interpreting which variables are important and how these form structures of feedback that can be valid explanations in the studied case. It is an iterative process of identifying the best fit with the stories of the empirical findings and representing the current hypothesis given current understanding. Hence, such a model is an evolving object towards higher levels of identified understanding and representation of the studied phenomena. Also, important to consider is that the relations in the CLD create possibility to draw upon a multitude of time-dependent scenarios, all existing in the same model representation, analysed in the subsequent section to the model description.

First, an overview of the themes mapped into the model is provided, followed by a description detailing the themes in the model. After the model description, the systems thinking model is analysed. How the model can facilitate further learning is elaborated later in the discussion.

**Figure 1:**
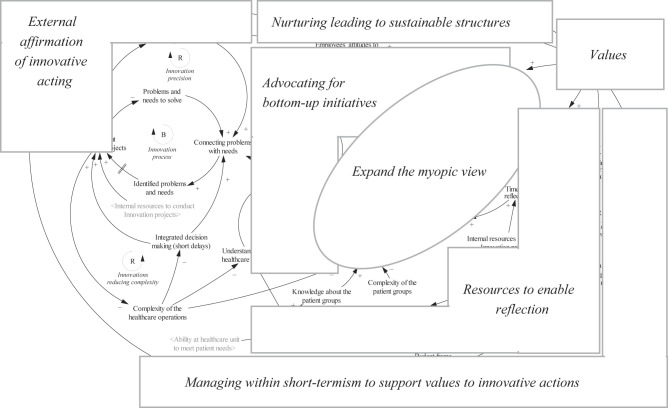
Model layout overview, including the empirically derived themes

Figure 1 provides an overview of how the empirical themes map with the developed CLD, represented by graphical objects of different shapes, which are also referred to as “parts” further on. The overview reveals that the themes do not fully cover the CLD, which has a higher resolution regarding describing a holistic unity and function between the themes. For instance, *Values* is represented by a small box in the overview, but manifest into all the other parts, elaborated below in the detailed model description.

### Detailing the themes in the model

**Figure 2:**
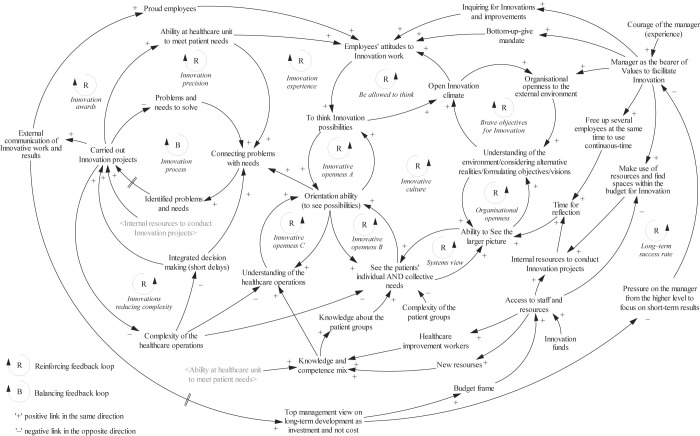
The main interpreted interactions between factors in the studied healthcare innovation system, using Vensim software

In the description of the themes in the model below the variable names and feedback loops are denoted in italics, where the reader can find its corresponding name in the model below in Figure 2 . It is recommended to study the model while reading the descriptions to enhance understanding.

*Values* : The variable *Manager as the bearer of Values to facilitate Innovation* attributes to the former manager of the unit, inherited by her successor, and the expressed values and acting out of her beliefs in what can result in nurturing an innovative culture. The result of deliberate and perseverant advocacy work to nurture the innovative work by the manager led to a *Long-term success rate* generated by *Carried out Innovation projects* . The several entities in the model affected by the acted-out *values* of the manager interact with all the themes. Therefore, each subsequent theme begins with how the values interact with them in their descriptions.

*Expand the myopic view* : First, *values* facilitated expanding the myopic view by growing the employees' appreciation for competencies beyond their health profession and permeating the lubrication of multiple processes in this part. Introducing contacts with the external environment allowed growing an *Organisational openness to the external environment* , which in due course led to increased *Understanding of the environment/considering alternative realities/formulating objectives/vision,* which reinforced the *Open Innovation climate* and set more and more *Brave objectives for Innovation.* Nurturing this openness made it possible to increase the *Ability to See the larger picture,* which generated even higher levels of understanding and brave objectives, reinforcing the *Organisational openness* feedback loop, where a vital enabler for the development was *Time for reflection.* Also, seeing the larger picture improved the *Systems view,* implying increased ability to *See the patients* *'* *individual AND collective needs* at once.

*Advocating for bottom-up initiatives* : By acting out desired shared *values,* the manager was *Inquiring for Innovations and improvements* and, together with the value represented by the variable *Bottom-up – give mandate* *,* it nurtured *Employees attitudes to Innovative work,* encouraging them *To think Innovation possibilities* from their current understanding; this empowered a trusted *Open Innovation climate,* reinforcing the behaviour of the feedback loop *Be allowed to think* . Hence, the *values* also nurtured the growth of the three feedback loops – *Innovative openness (A, B and C)* – where thinking innovation possibilities co-evolved with a reinforced system view on patients' individual and collective need. It also reinforced *Understanding of the healthcare operations* , but primarily empowered the *Orientation ability to see possibilities* , which was something the manager wanted to achieve, leading to creating a decentralised improvements culture. Hence, the variables and feedback processes in the *expan* *sion* *of* *the myopic view* and *advocating for bottom-up initiative* parts work tightly together to empower the complete reinforcing feedback loop *Innovative culture* .

*External affirmation of innovative acting* : How *values* interact with this part is not explicitly denoted in the model, but it was an active interaction from the former manager. The manager strongly encouraged *External communication of Innovative work and results* , from *Carried out Innovation projects,* to be presented by the employees themselves at multiple arenas, both externally and at the hospital. Internally at the unit, this was considered to create *Proud employees* and improved the *Employees* *'* *attitudes to Innovati* *ve* *work* , strengthening the *Innovative culture* loop, and nurtured employees' self-image into being “active and taking initiatives”. The external communication also led to softening *Top management view on long-term development and not cost* , which in due course was believed to affect both the view of *Budget frame* to eventually start considering including resources for innovative work inside the ordinary budget and to reduce the pressure of short-term results. However, this was merely emerging during the study and had not yet come into effect, so a delay mark is included on the causal link. Even if some noticeable change was reported, it had not resulted in concrete action beyond only praising the results; therefore, nurturing the *Long-term success rate* feedback loop is considered to require perseverance.

*Resources to enable reflection* : One important link where *values* explicitly had affected the view on resources was the belief in the importance of having *Time for reflection* on daily work and development projects to nurture the *Innovative culture* . It was acted out by deliberate planning of resources to *Free up several employees at the same time to use continuous time* for innovative work, which also made it necessary to *Make use of resources and find spaces within the budget for Innovation* , and to supply enough *Internal resources to conduct Innovation projects.* Supporting this process solely by internal means was considered possible, but too slow. So the manager initiated searching and applying for external and regional research funds for healthcare innovations projects, represented by *Innovation funds,* to enable *Access to staff and resources* outside the unit's *Budget frame.* This made it possible to utilise *Internal resources to conduct Innovation projects* by hiring substitutes to enable the ordinary staff to implement the innovative work. It also made it possible to involve *Healthcare improvement workers* and employ *New resources,* which broadened the *Knowledge and competence mix* , further strengthening the *Orientation ability* via some variables.

*Managing within short-termism to support values to innovative actions* : From the *values* perspective, these worked counter to the current short-term focussed top management paradigm. It also required nurturing the innovative culture undercover due to the lack of *Top management view on long-term development as investment and not cost* , as evident in the prominent *Pressure on the manager from the higher level to focus on short-term results* . Despite the less innovative culture at the hospital level, the unit manager could manifest her values and actions, requiring a high level of *Courage of the manager,* which came from experience and the internal beliefs in that applied strategies to nurture an *Innovative culture* would last in the long run.

*Nurturing leading to sustainable structures* : By *Inquiring for Innovations and improvements* regularly on staff meetings, which is ascribed to organisational structure, the enquiring worked together with the many above-mentioned acted-out shared values connected to *values.* The stated aim was to nurture the organisational culture through enabling employees seeing, thinking and performing innovations. It is found that the model focuses mainly on the elements nurturing the culture and provides less attention to any further organisational structural elements.

### Variables to complete the model

To present a complete CLD, some additional variables related to but not explicitly part of the presented themes were needed especially, regarding how the reported healthcare innovation processes were nurtured by the more and more innovative culture. Starting with an increased *Orientation ability* , generated by the progress of the *Innovative culture* , it also increased the ability of *Connecting problems with needs,* which leads to more opportunities possibly turning into *Identified problems and needs* and subsequently more *Carried out Innovation projects,* which is the observable output. The output, in turn, builds *Proud employees* in the *Innovation awards* feedback loop, which seemed to soften the short-term view maintained by the top management as mentioned before, and also reduces *Problems and needs to solve* as they get connected in the subsequent variable. Envisioning the *Problems and needs to solve* as a backlog of opportunities can support understanding the only balancing loop considered in the model termed *Innovation process.* A backlog can illustrate consuming the results of an *Innovative culture* by the *Innovation process,* which is eligible to release the economical and healthcare quality benefits. The dynamics generated from a balancing loop coupled with a reinforcing loop is characterised by an S-shaped behaviour ( Sterman, 2000 ). The effect of this is analysed in the next subsection. Before closing, some more variables should be considered. Another output from *Carried out Innovation projects* is the generated learning, which increases the *Ability at healthcare unit to meet patient needs* , which reinforces the *Innovation precision* of *Connecting problems with needs,* as well as generating *Innovation experience* , reinforcing the *Employees* *'* *attitudes to Innovati* *ve* *work* by experiences from *Carried out Innovation projects* via parts of the *Innovative culture* loop. Furthermore, an increased ability to meet patient needs is also having the effect of increasing the *Knowledge and competence mix* of the organisation, leading to improved prerequisites for strengthening the *Orientation ability* and in extension of the *Innovation culture* . Another aspect is the implementation effects from innovation projects that reduce complexity, lowering the *Complexity of the healthcare operations* . A less complex healthcare context has similar effects as increased knowledge, where lesser complexity potentially simplifies *Understanding of the healthcare operations* and made it easier to *See the patients* *'* *individual AND collective needs.* Less complex healthcare operations were considered to strengthen the *Integrated decision making,* which was pointed out in the interviews to facilitate the *Innovation process* by enabling managerial support on *Connecting problems with needs* and facilitated the implementation of *Carried out Innovation projects* .

### Analysing the systems thinking model

Two central dynamic forces can be distinguished in the model. Firstly, the network of reinforcing feedback that directly and indirectly supports the nurturing of an *Innovative culture* , and secondly, one balancing loop called *Innovation process* . The model represents the empirical findings in terms of *how* the auxiliary variables to these two main dynamic forces could be composed to create different needful preconditions and require active processes of interconnected nature to their support. Without being exhaustive and including all possible dynamics of the proposed model, which can serve as a vehicle for multiple reasoning around plausible dynamics, the subsequent analysis focuses on the interactions of accelerating the innovation processes by nurturing an innovative culture to help understand aspects of the healthcare innovation system.

Reinforcing feedback loops in systems either generate better and better or worse and worse behaviour. Having a network of coupled feedback loops, as appeared in the proposed model, gives rise to chains of dependencies amongst variables. And a chain is not stronger than its weakest link, resulting in the innovation system having possible locks between variables. It means the current situation of any healthcare context plays out differently, and if the precondition in any variable is low, it has a portion of restraining effects to the desired evolving dynamics, likely leading to several variables needing to be stimulated to start and generate better and better behaviour. Hence, it indicates that the preconditions to ignite an innovative journey need to be nurtured on a broad spectrum with the appropriate *values,* while a limited effort will likely be locked in. Given these characteristics, the *Innovative culture* is continuously changing with a certain inertia by path dependency towards higher, or lower, levels. Hence, even though innovation culture is a complex phenomenon of several hard-to-distinguish characteristics ( Schein, 2017 ), the model can be considered to conclude many important efforts found in the studied unit that need to be orchestrated to achieve desired change. From this analysis, perseverance to achieve improved healthcare operations and subsequent improvement for the patients has been identified as critical. Moreover, considering what composes negative or positive factors affecting the system behaviour, meaning what hinders or drives building capacity in the innovation system, the model could support realising they can be seen as forces on the same axis in the reinforcing feedback loops. Consequently, nurturing the advantageous dynamics through the reinforcing feedback loops creates a drive that can eventually overcome current hindrances. In effect, the lack of nurturing the desired *values* of an innovative culture slows or hinders the innovative processes to emerge, resulting in a lack of innovativeness. Potentially it defines what the opposite of an innovative culture is: no generative power in the innovation processes. Additionally, it can be assumed due to the interconnected networks of reinforcing feedback, that the activation of desired behaviour from nurturing culture eventually becomes manifested in individual employees constructing new improved organisational structures. And, eventually these new structures presumably become taken for granted as the new normal of the system actors, as was observed in the studied case. Hence, the mindsets and mental models of people in the system together with organisational structures act as delays, creating inertia in both directions, towards even further accelerating innovation processes, or towards the deterioration of the attained innovative culture leading to less future innovations.

Balancing feedback loops are goal oriented and drive system behaviour towards current system equilibrium which is defined by the complex network of interconnected variables. Connecting and reinforcing with balancing loops often generates non-linear S-shaped growth, where the net increase of the rate that represents the growth is dependent on the state of the system ( Sterman, 2000 ). In one sense, nurturing the *values* to achieve an innovative culture introduces multiple subtle goals of desired development to a higher set *value* to induce this change process. Meaning, in a higher resolution model these goal-oriented mechanisms could be represented by balancing feedback loops. However, in the model the *values* have only been represented as causal links from *Manager as the bearer of Values to facilitate Innovation.* Yet, the aspect of having a connected reinforcing and balancing loop in the resulting model may be central to the short- and long-term dynamic behaviour. A specific prediction of all interactions is impossible to conclude, but, in general, following analysis can be drawn. The pace of innovations in a poor innovative culture is initially slow in terms of trying to nurture an innovative culture, and with time, generated by the reinforcing feedbacks exponentially growing to its maximum pace, to thereafter decay in pace due to the difficulty of identifying new potentials to innovate. Such dynamics can be illustrated by the following example. Starting with a poor level of *Innovative culture* it is more likely to require much more perseverance to generate the reinforcing *Long-term success rate* required to ease the pressure on short-term results from upper management, which is often critical to support a budget and resources for doing more innovative work to realise the long-term gains. This is because it builds upon *Carried out Innovation projects,* which may be the effect of long-term dedicated innovative work throughout the organisation. Having blockings between long-term and short-term mechanisms that are dependent on each other's mutual development like this creates a long feedback loop until rewards are evident from the initial attempts of innovating. Reasonably, the longer the delay in this feedback process (between a manager interacting to invest and nurture *values* until seeing effects of an *Innovative culture* ) the more perseverance is likely to be required. Meanwhile, it is at risk that other significant intervention points (above-mentioned desired developments in variables of the reinforcing feedback network) in need of being nurtured and maintained are neglected, potentially leading to a worsened new condition of short-termism instead of nurturing an *Innovative culture.* However, as dedication to nurture *values* and a more *Innovative culture* grows, it creates a more beneficial condition of *Orientation ability* of the employees, and through the development of *Carried out Innovation projects* and increased *Innovation precision* , it is reasonable to identify; it becomes easier and easier to connect problems with needs. Simultaneously, given the complexity of healthcare operations, it is reasonable to assume the innovation system is subject to change and adaption over time as new conditions and disruptive external innovations take place, creating a stream of internal innovation possibilities in a specific healthcare unit. However, it is also reasonable that a healthcare unit with less complex context, such as the studied case, has different dynamics than other more complex contexts, as well as the implications of the multitudes of factors not considered in the study, such as legal and medical aspects for a specific context that possibly can limit or create potential to innovate for the specific innovation system. On this note, relating also, to the implication of negative and positive factors affects the system behaviour. A negative factor in a higher state of innovative culture that hinders current development may not be of interest in a lower state of innovative culture due to the many other hindering factors first in need of being overcome.

## Discussion and conclusions

This study is considered to contribute to the scarce literature of building knowledge and understanding about innovation in healthcare and aims to provide support for *how* culture may be important for healthcare innovation. This study openly reviews the process of creating a CLD over nurturing an innovative culture as the generator to achieve momentum in the innovation processes. The presented results underscore the difficulty of defining what constitutes an innovative culture ( Schein, 2017 ), but may still support communicating important factors that possibly contribute to its existence helpful to future change initiatives and research. One major contribution from applying the systems thinking approach has been to help generalise the identified patterns and structures found in the thematic analyses of the interviews, visualised by the presented CLD. Hence, further utilisation of the CLD may allow for a more explicit dialogue on ways to nurture desired *values* leading to an innovative culture in other settings. The generalised view, grasped by the CLD, is not considered limited to a specific context but has strived to expose the generic dynamics identified. Perhaps, the results are even possibly valuable to other research fields, by replacing the healthcare context specific variables to the ones of another application field.

In the model, an aim was to capture the pieces of described stories from the interviewees into a complete story of all phenomena together. The model aimed to provide a holistic view of the studied healthcare organisation's innovation processes, ranging from managerial values to its manifestation in improved results. Thus, the proposed CLD aimed to build a reasonable explanation of the observed phenomena by hypothesising the causal relations of the interconnected variables. Accordingly, the process of modelling the CLD supported piecing together the different stories into reasonable coherent explanations, where the character of specific pieces of information could be represented by variables and feedback structures.

Although, this research may offer new insights, it is important to acknowledge some limitations. Firstly, even if the proposed model expresses generic elements of the innovation system relatable to healthcare organisations, it is based on a limited study in a speech therapy unit at a regional hospital. Such unit has a more coherent work force compared to most other healthcare settings and less unplanned events that require higher levels of resilience and add more complexity. Secondly, despite being based on the empirical findings, the coherence of the CLD to represent observed phenomena is verified only by the team of researchers and their perceptions of the thematic analyses. And, in terms of designing models there are benefits of utilising face validation ( Sargent, 2013 ), where stakeholders and problem owners are confronted with the model to verify its relevance and can validate its coherence with experienced phenomena. However, this study was not a traditional modelling case where the setting was defined as a simulation study from the beginning, e.g. that of Linnéusson and Goienetxea Uriarte (2021) . Instead, it was in the process of the thematic analyses of the interviews; the usability of using the CLD tool grew with respect to enhance representation of the entirety of the results in a coherent procedure. Thus, verification of the model, to eliminate logical incoherencies was carried out carefully, while validation of the CLD with the studied unit was not conducted within this study. Furthermore, the limitations to successfully design a generic CLD, even if it has been strived for, is restricted by the study's inductive character using stories from only one healthcare unit. In all, these limitations restrict direct transferability of the findings and need to be acknowledged in future research. Nevertheless, it is our belief that presenting these results openly to the research community allows for scrutiny of the model composition and, in effect, supports reuse and modifications to suit different contexts, serving as a base for further improvements to uncover driving and hindering dynamics of innovation.

Even if the reviewed analysis of the CLD is not exhaustive, analysing the model may support explaining the dynamics of inertia when nurturing an innovative culture. The analysis has identified many of the existing delays attributed to the internal change processes in the mental models of those in the system. In particular, the aspect that the “innovative culture” consists of a reinforcing feedback loop in the model, and the “innovation process” consists of a balancing feedback loop, creates a system behaviour of an S-shaped growth ( Sterman, 2000 ), where exponential growth in the early phases is small, reaches a maximum and then declines to saturation. Together, these structural elements are believed to help explain *why* innovativeness may significantly differ in the same healthcare organisation ( Exton, 2010 ) and, importantly, highlight the need for perseverance in growing an innovative culture in the entrepreneurial phases of an innovation system.

Finally, due to the generalised description of the presented CLD its contribution as an explicit learning model ( Peters, 2014 ) for further applications in other local healthcare contexts may be stated. And, considering usability of the results, the contribution from this study to the larger multi-case research programme its part has contributed to developing a dialogue tool for assessing healthcare innovation systems at the hospital of the conducted study. Besides these contributions, further usage of the specific CLD could also be valuable to explore. This can be done by using the model to help exploring preconditions or important considerations for how to achieve successful innovation management at units carried out in modelling workshops. By using stepwise presentations of the CLD as basis for discussions, in settings with healthcare professionals, managers and modellers, it may facilitate the development of more unified mental models and shared values for a specific context. Such process has been studied to support the multi-professional knowledge repository ( Holmström *et al.* , 2021 ), and is expected helpful to uncover the unknown unawareness of their innovative culture and facilitate the development of new shared understanding ( Bååthe and Norbäck, 2013 ). Therefore, suitable future research in the trajectory of this study is also proposing multiple case studies utilising the generalised CLD as basis when analysing current innovation systems in different healthcare contexts to further explore its applicability to support individual and organisational learning. In such studies we can learn even more about *how* different starting conditions of innovative culture can be nurtured and learn more of the specific essential challenges existing at differently complex healthcare contexts. Nevertheless, judging upon the content of the CLD and its purpose to facilitate seeing hindering and driving dynamics of an innovative system in healthcare, it is believed, despite its limitations, it may support dialogue to strengthen local health practitioners to innovate in their local hospital unit, and thereby support innovative transformational change, improving community health and thus the overall health system ( Swanson *et al.* , 2012 ).

## References

[ref001] Acar , A.Z. and Acar , P. ( 2012 ), “ The effects of organizational culture and innovativeness on business performance in healthcare industry ”, Procedia – Social and Behavioral Sciences , Vol. 58 , pp. 683 - 692 .

[ref002] Andersson , T. , Cäker , M. , Tengblad , S. and Wickelgren , M. ( 2019 ), “ Building traits for organizational resilience through balancing organizational structures ”, Scandinavian Journal of Management , Vol. 35 No. 1 , pp. 36 - 45 .

[ref003] Atun , R. ( 2012 ), “ Health systems, systems thinking and innovation ”, Health Policy and Planning , Vol. 27 , pp. 4 - 8 . 10.1093/heapol/czs088 23014152

[ref004] Bååthe , F. and Norbäck , L.-E. ( 2013 ), “ Engaging physicians in organisational improvement work ”, Journal of Health, Organisation and Management , Vol. 27 No. 4 , pp. 479 - 497 . 10.1108/JHOM-02-2012-0043 24003633

[ref005] Best , A. , Berland , A. , Herbert , C. , Bitz , J. , van Dijk , M.W. , Krause , C. , Cochrane , D. , Noel , K. , Marsden , J. , McKeown , S. and Millar , J. ( 2016 ), “ Using systems thinking to support clinical system transformation ”, Journal of Health Organisation and Management , Vol. 30 No. 3 , pp. 302 - 323 . 10.1108/JHOM-12-2014-0206 27119388

[ref006] Braun , V. and Clarke , V. ( 2006 ), “ Using thematic analysis in psychology ”, Qualitative Research in Psychology , Vol. 3 No. 2 , pp. 77 - 101 .

[ref007] Carmo Caccia-Bava , M. , Guimaraes , T. and Harrington , S. ( 2006 ), “ Hospital organization culture, capacity to innovate and success in technology adoption ”, Journal of Health Organization and Management , Vol. 20 No. 3 , pp. 194 - 217 . 16869354 10.1108/14777260610662735

[ref008] Chang , A. , Ogbuoji , O. , Atun , R. and Verguet , S. ( 2017 ), “ Dynamic modeling approaches to characterize the functioning of health systems: a systematic review of the literature ”, Social Science and Medicine , Vol. 194 , pp. 160 - 167 . 29100141 10.1016/j.socscimed.2017.09.005

[ref009] Chughtai , S. and Blanchet , K. ( 2017 ), “ Systems thinking in public health: a bibliographic contribution to a meta-narrative review ”, Health Policy and Planning , Vol. 32 No. 4 , pp. 585 - 594 . 28062516 10.1093/heapol/czw159

[ref010] Day-Duro , E. , Lubitsh , G. and Smith , G. ( 2020 ), “ Understanding and investing in healthcare innovation and collaboration ”, Journal of Health Organization and Management , Vol. 34 No. 4 , pp. 469 - 487 . 10.1108/JHOM-07-2019-0206 32250574

[ref011] Dopson , S. , Fitzgerald , L. and Ferlie , E. ( 2008 ), “ Understanding change and innovation in healthcare settings: reconceptualizing the active role of context ”, Journal of Change Management , Vol. 8 Nos 3-4 , pp. 213 - 231 .

[ref012] Elias , A. ( 2021 ), “ Kerala's innovations and flexibility for covid-19 recovery: storytelling using systems thinking ”, Global Journal of Flexible Systems Management , Vol. 22 No. 1 , pp. 33 - 43 . 10.1007/s40171-021-00268-8 PMC8021297 40477226

[ref013] Evans , J. and Baker , R. ( 2012 ), “ Shared mental models of integrated care: aligning multiple stakeholder perspectives ”, Journal of Health Organisation and Management , Vol. 26 No. 6 , pp. 713 - 736 . 10.1108/14777261211276989 23252323

[ref014] Exton , R. ( 2010 ), “ Enterprising health: creating the conditions for entrepreneurial behaviour as a strategy for effective and sustainable change in health services ”, Journal of Health Organisation and Management , Vol. 24 No. 5 , pp. 459 - 479 . 10.1108/14777261011070493 21033640

[ref015] Forrester , J. ( 1961 ), Industrial Dynamics , MIT Press , Cambridge, Massachusetts .

[ref016] Haynes , A. , Garvey , K. , Davidson , S. and Milat , A. ( 2020 ), “ What can policy-makers get out of systems thinking? Policy partners' experiences of a systems-focused research collaboration in preventive health ”, International Journal of Health Policy and Management , Vol. 9 No. 2 , pp. 65 - 76 . 32124590 10.15171/ijhpm.2019.86 PMC7054651

[ref017] Holmström , P. , Hallberg , S. , Björk-Eriksson , T. , Lindberg , J. , Olsson , C. , Bååthe , F. and Davidsen , P. ( 2021 ), “ Insights gained from a systematic reanalysis of a successful model-facilitated change process in health care ”, Systems Research and Behavioral Science , Vol. 38 No. 2 , pp. 204 - 214 .

[ref018] Keller , C. , Edenius , M. and Lindblad , S. ( 2013 ), “ Open service innovation in health care: what can we learn from open innovation communities? ”, in Eriksson Lundström , J. , Wiberg , M. , Hrastinski , S. , Edenius , M. and Ågerfalk , P. (Eds), Managing Open Innovation Technologies , Springer , Berlin and Heidelberg , pp. 239 - 251 .

[ref019] Kwamie , A. , Ha , S. and Ghaffar , A. ( 2021 ), “ Applied systems thinking: unlocking theory, evidence and practice for health policy and systems research ”, Health Policy and Planning , Vol. 36 No. 10 , pp. 1715 - 1717 . 34131699 10.1093/heapol/czab062 PMC8597965

[ref020] Larisch , L.-M. , Amer-Wåhlin , I. and Hidefjäll , P. ( 2016 ), “ Understanding healthcare innovation systems: the Stockholm region case ”, Journal of Health Organization and Management , Vol. 30 No. 8 , pp. 1221 - 1241 . 27834601 10.1108/JHOM-04-2016-0061

[ref021] Leslie , H.H. , Hirschhorn , L.R. , Marchant , T. , Doubova , S.V. , Gureje , O. and Kruk , M.E. ( 2018 ), “ Health systems thinking: a new generation of research to improve healthcare quality ”, PLoS Medicine , Vol. 15 No. 10 , pp. 5 - 8 . 10.1371/journal.pmed.1002682 PMC6207294 30376581

[ref022] Linnéusson , G. , Ng , A. and Aslam , T. ( 2018 ), “ Towards strategic development of maintenance and its effects on production performance by using system dynamics in the automotive industry ”, International Journal of Production Economics , Vol. 200 , pp. 151 - 169 .

[ref023] Linnéusson , G. , Ng , A. and Aslam , T. ( 2020 ), “ A hybrid simulation-based optimization framework supporting strategic maintenance development to improve production performance ”, European Journal of Operational Research , Vol. 281 No. 2 , pp. 402 - 414 .

[ref024] Linnéusson , G. and Goienetxea Uriarte , A. ( 2021 ), “ Analyzing closer care strategies for elderly patients: experience and reflections from modeling with system dynamics ”, in Fakhimi , M. , Robertson , D. and Boness , T. (Eds), Proceedings of the Operational Research Society Simulation Workshop 2021 , pp. 117 - 126 .

[ref025] Mannion , R. and Davies , H. ( 2018 ), “ Understanding organisational culture for healthcare quality improvement ”, BMJ , Vol. 363 , pp. 1 - 4 . 10.1136/bmj.k4907 PMC6260242 30487286

[ref026] Øvretveit , J. , Andreen-Sachs , M. , Carlsson , J. , Gustafsson , H. , Hansson , J. , Keller , C. , Lofgren , S. , Mazzocato , P. , Tolf , S. and Brommels , M. ( 2012 ), “ Implementing organisation and management innovations in Swedish healthcare: lessons from a comparison of 12 cases ”, Journal of Health Organization and Management , Vol. 26 No. 2 , pp. 237 - 257 . 22856178 10.1108/14777261211230790

[ref027] Peters , D. ( 2014 ), “ The application of systems thinking in health: why use systems thinking? ”, Health and Quality of Life Outcomes , Vol. 12 No. 1 , pp. 1 - 6 . 10.1186/1478-4505-12-51 PMC4245196 25160707

[ref028] Rusoja , E. , Haynie , D. , Sievers , J. , Mustafee , N. , Nelson , F. , Reynolds , M. , Sarriot , E. , Swanson , R.C. and Williams , B. ( 2018 ), “ Thinking about complexity in health: a systematic review of the key systems thinking and complexity ideas in health ”, Journal of Evaluation in Clinical Practice , Vol. 24 No. 3 , pp. 600 - 606 . 29380477 10.1111/jep.12856

[ref029] Sargent , R.G. ( 2013 ), “ Verification and validation of simulation models ”, Journal of Simulation , Vol. 7 No. 1 , pp. 12 - 24 .

[ref030] Sarkies , M. , Robinson , S. , Ludwick , T. , Braithwaite , J. , Nilsen , P. , Aarons , G. , Weiner , B.J. and Moullin , J. ( 2021 ), “ Understanding implementation science from the standpoint of health organisation and management: an interdisciplinary exploration of selected theories, models and frameworks ”, Journal of Health Organization and Management , Vol. 35 No. 7 , pp. 782 - 801 .

[ref031] Saulnier , D.D. , Blanchet , K. , Canila , C. , Muñoz , D.C. , Dal Zennaro , L. , de Savigny , D. , Durski , K.N. , Garcia , F. , Grimm , P.Y. , Maceira , D. , Marten , R. , Peytremann- Bridevaux , I. , Poroes , C. , Ridde , V. , Seematter , L. , Stern , B. , Suarez , P. , Teddy , G. , Wernli , D. , Wyss , K. and Tediosi , F. ( 2021 ), “ A health systems resilience research agenda: moving from concept to practice ”, BMJ Global Health , Vol. 6 No. 8 , e006779 . 10.1136/bmjgh-2021-006779 PMC8344286 34353820

[ref032] Savory , C. and Fortune , J. ( 2015 ), “ From translational research to open technology innovation systems ”, Journal of Health Organization and Management , Vol. 29 No. 2 , pp. 200 - 220 . 25800333 10.1108/JHOM-01-2013-0021

[ref033] Schein , E. ( 2017 ), Organizational Culture and Leadership , 5th ed. , Wiley , Hoboken .

[ref034] Senge , P. and Sterman , J. ( 1992 ), “ Systems thinking and organizational learning: acting locally and thinking globally in the organization of the future ”, European Journal of Operational Research , Vol. 59 No. 1 , pp. 137 - 150 .

[ref035] Stalter , A. and Mota , A. ( 2018 ), “ Using systems thinking to envision quality and safety in healthcare ”, Nursing Management , Vol. 49 No. 2 , pp. 32 - 39 . 10.1097/01.NUMA.0000529925.66375.d0 29381534

[ref036] Sterman , J. ( 2000 ), Business Dynamics: Systems Thinking and Modeling for a Complex World , Irwin McGraw-Hill , Boston, Massachusetts .

[ref037] Swanson , R.C. , Cattaneo , A. , Bradley , E. , Chunharas , S. , Atun , R. , Abbas , K.M. , Katsaliaki , K. , Mustafee , N. , MeierMeier , B.M. and Best , A. ( 2012 ), “ Rethinking health systems strengthening: key systems thinking tools and strategies for transformational change ”, Health Policy and Planning , Vol. 27 , Suppl. 4, pp. 54 - 61 . 10.1093/heapol/czs090 PMC3529625 23014154

[ref038] Thune , T. and Mina , A. ( 2016 ), “ Hospitals as innovators in the health-care system: a literature review and research agenda ”, Research Policy , Vol. 45 No. 8 , pp. 1545 - 1557 .

[ref039] Waldman , J.D. ( 2007 ), “ Thinking systems need systems thinking ”, Systems Research and Behavioral Science , Vol. 24 No. 3 , pp. 271 - 284 .

[ref040] Wilkinson , J. , Goff , M. , Rusoja , E. , Hanson , C. and Swanson , R.C. ( 2018 ), “ The application of systems thinking concepts, methods, and tools to global health practices: an analysis of case studies ”, Journal of Evaluation in Clinical Practice , Vol. 24 No. 3 , pp. 607 - 618 . 29152819 10.1111/jep.12842

[ref500] WMA ( 2008 ), available at: https://www.wma.net/what-we-do/medical-ethics/declaration-of-helsinki/doh-oct2008/ .

[ref041] Wolstenholme , E. ( 2004 ), “ Using generic system archetypes to support thinking and modelling ”, System Dynamics Review , Vol. 20 No. 4 , pp. 341 - 356 .

